# Genetic testing of hereditary antithrombin deficiency in a large US pedigree using saliva samples

**DOI:** 10.1111/ijlh.13390

**Published:** 2020-11-21

**Authors:** René Mulder, Karina Meijer, Michaël V. Lukens

**Affiliations:** ^1^ Department of Laboratory Medicine University Medical Center Groningen University of Groningen Groningen The Netherlands; ^2^ Division of Haemostasis and Thrombosis Department of Haematology University Medical Center Groningen University of Groningen Groningen The Netherlands

Dear Editors,

Antithrombin is the most important physiological inhibitor of coagulation. Antithrombin predominantly inhibits thrombin and factor Xa. Interestingly, the inhibitory function of antithrombin is remarkably slow due to its repressed reactivity state. The function of antithrombin is significantly enhanced by heparin or heparan sulfates.

Antithrombin deficiency is a rare autosomal dominant disorder, characterized by a fourteen‐fold increased risk of venous thromboembolism (VTE).^1^ Antithrombin deficiency can be classified into type I (quantitative defect) and type II (qualitative defect). Type II deficiencies can be further subdivided into type II RS (reactive site), type II HBS (heparin binding site), and type II PE (pleiotropic effects).^2^

Existing evidence suggests that in most cases antithrombin deficiency can be explained by mutations in its gene *SERPINC1*.^3^ To date, about 300 *SERPINC1* gene mutations have been reported to be associated with antithrombin deficiency.^4^ Most are point mutations or small insertion/deletion mutations.

At present, blood samples are favored for obtaining high‐quality DNA; however, DNA can also be obtained by collecting saliva, which creates benefits as its painless, noninvasive sample collection, ideal for use with children or patients that will not comply with blood collections, or have no direct access to a specialized laboratory.

This study investigated the genetic background of a large American pedigree with hereditary antithrombin deficiency using saliva samples.

In 2019, our Thrombosis & Haemostasis Center was contacted by a family member with the question whether we could help elucidate the antithrombin deficiency that runs in the family. As we have a special interest in antithrombin deficiency,^3^ we agreed to do so. Because our medical center is located in the Netherlands and participants live in United States of America, we choose to collect saliva instead of blood samples.

Each participant received a questionnaire, informed consent, and saliva Oragene‐DNA collection kit (ref OG‐250) (DNA Genotek, Canada). Forms and collection kit were shipped back at room temperature to our center in the Netherlands using UN3373 biological sample packaging solution from DHL. Our study enrolled adult subjects (≥18 years) after written informed consent. Genomic DNA was obtained from saliva. Prior to DNA isolation, each saliva container was gently shaken for at least 10 seconds after which they were incubated in a stove at 60 degrees for at least 1 hour. Next, after mixing, 200 μL saliva was used for isolation using the Qiacube^®^ system (QIAGEN). The concentration and purity of isolated genomic DNA was analyzed using the NanoDrop™ 2000 spectrophotometer (Thermo Fisher Scientific). The quality of isolated DNA was analyzed by agarose gel electrophoresis. Furthermore, we determined the human and bacterial DNA content by means of PCR amplification using previously published primer sets specific for human beta‐globin or bacterial 16s rRNA.^5^

Bi‐directional Sanger sequencing analysis of all 7 exons and flanking introns of *SERPINC1* gene was performed to detect sequence variations in the parent. If a sequence variation was found in the parent, all relatives were tested for that specific variant.

Isolated DNA was of high molecular weight and of similar quantity with the exception of sample 15 (Table S1 and Figure S1A), which showed signs of degradation as depicted by the smear. Furthermore, contamination of saliva DNA with bacterial DNA is not uncommon. Therefore, we investigated whether bacterial DNA was present after DNA extraction. With the exception of slightly lighter band for beta‐globin for sample 15, we did not observe any differences for all other samples between the DNA samples regarding the content of beta‐globin and 16s rRNA (Figure S1B,C).

**Figure 1:**
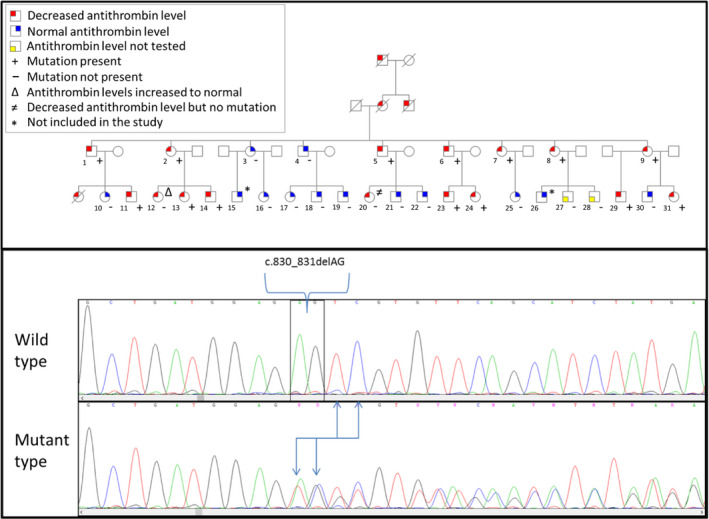
Upper part depicts included family. Lower part depicts chromatograms for sequencing results of wild type and mutant type. The location and effect of the mutation are highlighted

In total, we included 29 family members of which 16 were classified as antithrombin deficient, another 11 were not deficient and of the remainder the antithrombin levels were not measured (Table 1 and Figure 1). This was a high response rate considering the fact that we send 31 family members an envelope to participate in this study.

**Table 1:** Antithrombin levels

Participant	Levels	Participant	Levels
1	Level decreased^a^	17	115%
2	67%	18	Normal level
3	Normal level	19	Normal level
4	Normal level	20	Level decreased^a^
5	50%	21	Normal level
6	54%	22	Normal level
7	62%	23	Level decreased^a^
8	70%	24	31%
9	Level decreased^a^	25	Normal level
10	Normal level	26	Normal level
11	39%	27	Not tested
12	Decreased > normal	28	Not tested
13	67%	29	56%
14	Level decreased^a^	30	Normal level
15	Normal level	31	31%
16	Normal level		

^a^ Level unknown. Participant numbers correspond to Figure 1.

In order to elucidate the molecular background of hereditary antithrombin deficiency in a large American pedigree, we performed bi‐directional sequencing of all 7 exons and flanking introns, and found a small heterozygous deletion in exon 5 of *SERPINC1* gene (NM_000488:c.830_831del) (Figure 1). Furthermore, we identified 2 synonymous variants: c.981A > G (rs5877) and c.1011A > G (rs5878). The rs5877(C) allele was correlated with rs5878(C) allele and both had an allele frequency of 39.7%.

Antithrombin deficiency has been shown to be explained by multiple mutations in its gene. Among these mutations, missense and deletions are the most common. In this study by using bi‐directional sequencing, we found a heterozygous deletion in exon 5 of *SERPINC1* gene (NM_000488:c.830_831del) in large American pedigree with hereditary antithrombin deficiency. Based on the sequence string, this mutation has 3 additional equivalent positions. Even though we performed bi‐directional sequencing, we cannot exactly say what the position of the mutation should be. However, according to the HGVS recommendation we described the mutation at the first genomic position (chr1: 173879012). The mutation causes a frameshift leading to a premature termination codon (PTC) (NM_000488:p.(Glu277Valfs*20) (Figure 1). PTCs could result in mRNA degradation by nonsense‐mediated mRNA decay or truncated protein synthesis. This mutation segregated with phenotype antithrombin deficiency in all but two family members. Participant number 12 who reported low levels that increased to normal over time did not carry the mutation and the low levels of antithrombin previously reported could be due to a laboratory error. The latter may also be the case for the other participant (number 23) who did not carry the mutation or there was an another cause of a temporarily acquired antithrombin deficiency, such as administration of unfractionated heparin, liver disease, nephrotic syndrome, or a state of diffuse intravascular coagulation at the time of antithrombin measurement. Unfortunately, this information is not available to further explain the reported temporarily lower antithrombin levels.

The mutation we found has been described before by 2 separate groups who searched for variants causing hereditary antithrombin deficiency.^6^, ^7^ Both studies confirmed the segregation with type I antithrombin deficiency. The question whether these 2 studies describe results from far descendants of our family remains to be answered. However, based on the migration flow of people from Italian descent (origin of current family), it is tempting to assume a familial relationship with these studied participants.

To the best of our knowledge, this is the first time *SERPINC1* sequencing analyses have been done on DNA isolated from human saliva samples obtained from antithrombin‐deficient individuals. With the exception of sample 15, the results from DNA isolates were of good quality and sufficiently high concentration (Table S1). These results are comparable to a previous study.^5^

Taken together, we were able to set‐up an easy extraction method for the isolation of DNA from saliva with high quality and quantity. Moreover, using saliva samples instead of EDTA or citrate blood creates benefits as its painless, noninvasive sample collection, ideal for use with children or patients that will not comply with blood collections. Furthermore, samples can be mailed, remain stable for 5 years at room temperature, reducing transportation and storage costs and ease family and segregation analysis for patients and families with no direct access to specialized laboratories and centers for genetic antithrombin testing.

In conclusion, we herein provide evidence for the first time that *SERPINC1* gene analysis can be performed on saliva samples and that the *SERPINC1* mutation c.830_831del mutation is a definitely causative mutation for antithrombin deficiency type I. These results will add to our understanding of the molecular basis for antithrombin deficiency.

## CONFLICT OF INTEREST

Dr Meijer reports receiving research grants from Pfizer, Bayer, and Sanquin, lecturing fees from Bayer, Sanquin, Boehringer Ingelheim, BMS, and Aspen and consulting fees from Uniqure. All fees are paid to the institute.

## Supporting information

Supplementary Material

## Data Availability

Data sharing not applicable to this article as no datasets were generated or analysed during the current study.

## References

[1] Croles FN , Borjas‐Howard J , Nasserinejad K , Leebeek FWG , Meijer K . Risk of venous thrombosis in antithrombin deficiency: a systematic review and bayesian meta‐analysis. Semin Thromb Hemost. 2018;44:315‐326. 29452444 10.1055/s-0038-1625983

[2] Crowther MA , Kelton JG . Congenital thrombophilic states associated with venous thrombosis: a qualitative overview and proposed classification system. Ann Intern Med. 2003;138:128‐134. 12529095 10.7326/0003-4819-138-2-200301210-00014

[3] Mulder R , Croles FN , Mulder AB , Huntington JA , Meijer K , Lukens MV . SERPINC1 gene mutations in antithrombin deficiency. Br J Haematol. 2017;178:279‐285. 28317092 10.1111/bjh.14658

[4] Stenson PD , Ball EV , Mort M , et al. Human Gene Mutation Database (HGMD): 2003 update. Hum Mutat. 2003;21:577‐581. 12754702 10.1002/humu.10212

[5] Poehls UG , Hack CC , Ekici AB , et al. Saliva samples as a source of DNA for high throughput genotyping: an acceptable and sufficient means in improvement of risk estimation throughout mammographic diagnostics. Eur J Med Res. 2018;23:20. 29703267 10.1186/s40001-018-0318-9 PMC5921411

[6] Grundy CB , Thomas F , Millar DS , et al. Recurrent deletion in the human antithrombin III gene. Blood. 1991;78:1027‐1032. 1868237

[7] Kjaergaard AD , Larsen OH , Hvas AM , Nissen PH . SERPINC1 variants causing hereditary antithrombin deficiency in a Danish population. Thromb Res. 2019;175:68‐75. 30721820 10.1016/j.thromres.2019.01.022

