# Supplementary material for: Genetic testing of hereditary antithrombin deficiency in a large US pedigree using saliva samples
Source: Int J Lab Hematol. 2020 Nov 21;43(3):e101–3. doi: 10.1111/ijlh.13390 (PMC8246558; doi:10.1111/ijlh.13390)
Supplement: Supplementary file 1 — Supplementary Material [file IJLH-43-e101-s001.docx]

Supplementary


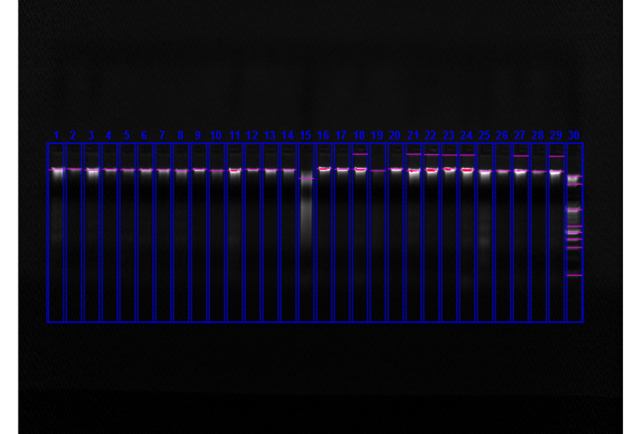

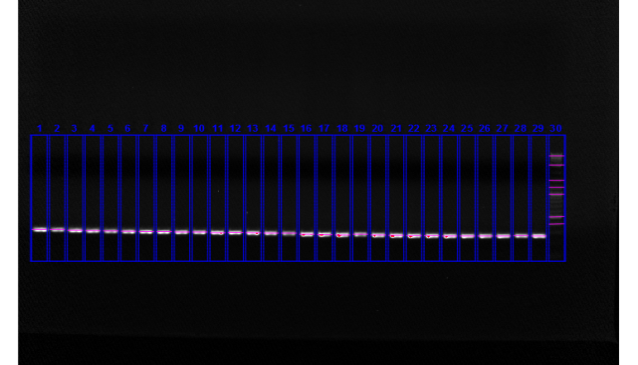

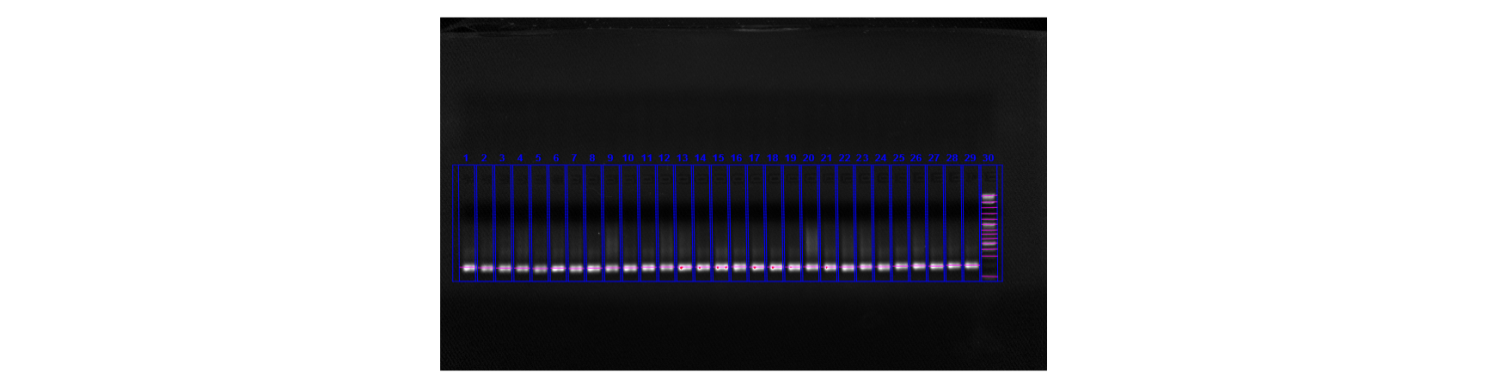


Genomic DNA

Human beta-globin

Bacterial 16s rRNA

A

B

C

Figure 1. Assessing quality of human and bacterial DNA for 29 samples. The amplified fragments were visualized by gel electrophoresis. Ten microlitres of amplified fragment was mixed with 2 μl of loading buffer. After mixing 10 μl was loaded onto a 1.5% agarose gel containing 0.08% ethidium bromide solution (Sigma-Aldrich, United States). Gel image of isolated DNA was performed with Gel Doc EZ imaging system (Bio-Rad, United States) together with Image Lab^tm^ software version 6.0 (Bio-Rad, United States). A 100 bp DNA ladder was used. A. Gel electrophoresis of isolated human genomic DNA from saliva. B. Amplification of human beta-globin. C. Amplification of Bacterial 16s rRNA.

Table 1. DNA concentrations and purity measured by the NanoDrop 2000.

| **Sample** | **Part.nr** | **Conc (ng/ul)** | **260/280** |  | **Sample** | **Part.nr** | **Conc (ng/ul)** | **260/280** |
| --- | --- | --- | --- | --- | --- | --- | --- | --- |
| 1 | 29 | 50 | 1.8 |  | 16 | 17 | 30 | 1.8 |
| 2 | 1 | 12 | 1.7 |  | 17 | 3 | 21 | 1.7 |
| 3 | 12 | 51 | 1.8 |  | 18 | 4 | 38 | 1.8 |
| 4 | 20 | 29 | 1.7 |  | 19 | 5 | 12 | 1.8 |
| 5 | 2 | 23 | 1.8 |  | 20 | 27 | 16 | 1.9 |
| 6 | 11 | 25 | 1.8 |  | 21 | 8 | 50 | 1.8 |
| 7 | 24 | 21 | 1.8 |  | 22 | 28 | 35 | 1.9 |
| 8 | 13 | 30 | 1.8 |  | 23 | 16 | 34 | 1.8 |
| 9 | 21 | 16 | 1.9 |  | 24 | 6 | 53 | 1.9 |
| 10 | 9 | 13 | 1.8 |  | 25 | 30 | 82 | 1.9 |
| 11 | 31 | 41 | 1.9 |  | 26 | 14 | 21 | 1.9 |
| 12 | 10 | 9 | 1.7 |  | 27 | 18 | 57 | 1.9 |
| 13 | 25 | 16 | 1.9 |  | 28 | 19 | 17 | 1.7 |
| 14 | 22 | 46 | 2 |  | 29 | 23 | 47 | 1.9 |
| 15 | 7 | 171 | 1.9 |  |  |  |  |  |
